# Glioma in Schizophrenia: Is the Risk Higher or Lower?

**DOI:** 10.3389/fncel.2018.00289

**Published:** 2018-09-03

**Authors:** Xingchun Gao, Yajing Mi, Na Guo, Hao Xu, Pengtao Jiang, Ruisan Zhang, Lixian Xu, Xingchun Gou

**Affiliations:** ^1^Shaanxi Key Laboratory of Brain Disorders & Institute of Basic Medical Sciences & Institute of Basic and Translational Medicine, Xi’an Medical University, Xi’an, China; ^2^State Key Laboratory of Military Stomatology, Department of Anesthesiology, School of Stomatology, The Fourth Military Medical University, Xi’an, China

**Keywords:** schizophrenia, glioma, protection, DISC1, miRNA

## Abstract

Whether persons with schizophrenia have a higher or lower incidence of cancer has been discussed for a long time. Due to the complex mechanisms and characteristics of different types of cancer, it is difficult to evaluate the exact relationship between cancers and schizophrenia without considering the type of tumor. Schizophrenia, a disabling mental illness that is now recognized as a neurodevelopmental disorder, is more correlated with brain tumors, such as glioma, than other types of tumors. Thus, we mainly focused on the relationship between schizophrenia and glioma morbidity. Glioma tumorigenesis and schizophrenia may share similar mechanisms; gene/pathway disruption would affect neurodevelopment and reduce the risk of glioma. The molecular defects of disrupted-in-schizophrenia-1 (DISC1), P53, brain-derived neurotrophic factor (BDNF) and C-X-C chemokine receptors type 4 (CXCR4) involved in schizophrenia pathogenesis might play opposite roles in glioma development. Many microRNAs (miRNAs) such as miR-183, miR-9, miR-137 and miR-126 expression change may be involved in the cross talk between glioma prevalence and schizophrenia. Finally, antipsychotic drugs may have antitumor effects. All these factors show that persons with schizophrenia have a decreased incidence of glioma; therefore, epidemiological investigation and studies comparing genetic and epigenetic aberrations involved in both of these complex diseases should be performed. These studies can provide more insightful knowledge about glioma and schizophrenia pathophysiology and help to determine the target/strategies for the prevention and treatment of the two diseases.

## Introduction

Early in 1909 (Commissioners in Lunacy for England and Wales, 1909), researchers first proposed a hypothesis that cancer risk decreased in persons with schizophrenia. Since then, numerous cohort studies have been performed to compare cancer incidence rates in persons with schizophrenia with the general population. However, conflicting results have been found; some studies showed a relatively lower risk of cancer in persons with schizophrenia (Lawrence et al., 2000; Cohen et al., 2002; Ji et al., 2013). In particular, Li et al. (2018) reported that the overall cancer incidence among patients with schizophrenia was slightly decreased (RR = 0.90, 95% confidence interval (CI) 0.81–0.99) in an updated meta-analysis of 16 cohort studies in 2018. However, other studies demonstrated a rather higher cancer risk in persons with schizophrenia (Lichtermann et al., 2001; Hippisley-Cox et al., 2007; McGinty et al., 2012). To date, it is still difficult to determine whether schizophrenia is a tumor-promoting factor or a suppressive factor. Researchers tried to give some reasonable explanations for this “century puzzle”: Preti and Wilson (2011) proposed that smoking, alcohol use, sex and lifestyle difference could affect the diagnosis of cancer among persons with schizophrenia. In addition, the antipsychotic therapy itself may have indirect anticancer effects which contribute to the reduced cancer risk in persons with schizophrenia (Dalton et al., 2006; Preti and Wilson, 2011). Genetic explanations showed that the schizophrenia genes may inhibit tumorigenesis (Park et al., 2004; Shi et al., 2008; Ozbey et al., 2011).

Different types of tumor have different pathogenesis characteristics, and thus, it is hard to generally evaluate the cancer risk in persons with schizophrenia without considering distinct cancer types (Table 1). In fact, numerous epidemiological investigations showed increased risk of breast cancer in people with schizophrenia (Grinshpoon et al., 2005; Hippisley-Cox et al., 2007; Carlsen et al., 2008), but decreased risk of prostate cancer (Grinshpoon et al., 2005; Ji et al., 2013; Li et al., 2018; Table 1). As a disabling mental illness that seems to originate from a disorder of brain development (Owen et al., 2016), schizophrenia is thought to be closely related to brain cancer such as glioma. Hence, we herein mainly focused on the relationship between glioma and schizophrenia. In this review article, we will compare the mechanisms of the glioma tumorigenesis and schizophrenia and try to determine whether the glioma risk in schizophrenia is higher or lower.

**Table 1:** Summary of the relationship between the cancer risk and schizophrenia.

Cancer type	Relationship	References
Bladder cancer	Null relationship	Lin et al. (2013)
Bone cancer	Null relationship	Ji et al. (2013)
Brain cancer including glioma	Decreased	Grinshpoon et al. (2005) and Wang et al. (2017)
	Null relationship	Lin et al. (2013)
	Increased for men during the first follow-up year	Lawrence et al. (2000) and Dalton et al. (2005)
Breast cancer	Increased	Grinshpoon et al. (2005); Hippisley-Cox et al. (2007) and
		Lin et al. (2013)
	Increased in female patients	Catts et al. (2008); Ji et al. (2013) and McGinty et al. (2012)
	Null relationship	Li et al. (2018)
Colon cancer	Increased	Hippisley-Cox et al. (2007) and McGinty et al. (2012)
	Decreased	Ji et al. (2013) and Li et al. (2018)
	Null relationship	Lawrence et al. (2000) and Lin et al. (2013)
Connective tissue cancer	Null relationship	Ji et al. (2013)
Corpus uteri cancer	Increased	Grinshpoon et al. (2005)
Endocrine glands cancer	Null relationship	Ji et al. (2013)
Endometrium cancer	Null relationship	Ji et al. (2013)
Esophagus cancer	Decreased in male patients	Ji et al. (2013)
Gastroesophageal cancer	Null relationship	Hippisley-Cox et al. (2007)
Genitournary cancer	Increased significantly	Lin et al. (2013)
Kidney cancer	Null relationship	Lin et al. (2013)
	Decreased in female patients	Ji et al., 2013
Leukemia	Null relationship	Lawrence et al. (2000) and Lin et al. (2013)
	Decreased	Ji et al. (2013)
Liver cancer	Null relationship	Lin et al. (2013)
	Decreased in male	Ji et al. (2013)
Lung cancer	Increased	Grinshpoon et al. (2005) and McGinty et al. (2012)
	Null relationship	Lawrence et al. (2000) and Lin et al. (2013)
	Increased significantly in female patients	Li et al. (2018)
	Decreased in male patients	Ji et al. (2013)
Lymphoma	Null relationship	Lawrence et al. (2000) and Lin et al. (2013)
Non-hodgkin lymphoma	Decreased	Ji et al. (2013)
melanoma	Null relationship	Lawrence et al. (2000)
	Decreased significantly in female patients	Grinshpoon et al. (2005)
	Decreased	Ji et al. (2013)
Myeloma	Decreased	Ji et al. (2013)
Nervous system cancer	Decreased in female patients	Ji et al. (2013)
Pancreas cancer	Increase in male patients	Lawrence et al. (2000)
	Decreased	Ji et al. (2013)
Prostate cancer	Null relationship	Lawrence et al. (2000); Hippisley-Cox et al. (2007); McGinty et al. (2012)
		and Lin et al. (2013)
	Decreased	Grinshpoon et al. (2005); Ji et al. (2013) and Li et al. (2018)
Rectal cancer	Null relationship	Hippisley-Cox et al. (2007)
	Decreased	Ji et al. (2013)
Respiratory cancer	Decreased	Hippisley-Cox et al. (2007)
Stomach cancer	Null relationship	Lawrence et al. (2000); Lin et al. (2013) and Li et al. (2018)
	Decreased	Ji et al. (2013)
Testis cancer	Null relationship	Ji et al. (2013)
Thyroid gland cancer	Decreased in male patients	Ji et al. (2013)

## Persons With Schizophrenia Have Lower Risk of Gliomas?

Grinshpoon et al. (2005) reported that the standardized incidence ratios (SIRs) were statistically lower for cancer in the brain among men with schizophrenia (0.56, 95% CI 0.32–0.81). Dalton et al. (2005) found an increased brain cancer risk for men during the first follow-up year among persons with schizophrenia, but Lin et al. (2013) did not get similar results. As a result, the relationship between brain cancer risk and schizophrenia is still a puzzle. The brain cancers include the primary, secondary and extraparenchymal tumors (Brandão, 2016). We are interested in glioma, which is the most common and aggressive type of primary brain tumor (Gao et al., 2014, 2015). Previous epidemiological studies showed that persons with schizophrenia may less likely to suffer from gliomas (Grinshpoon et al., 2005; Wang et al., 2017). Although there were few epidemiological investigations about glioma risk and schizophrenia, many studies indicated that mechanisms involved in schizophrenia pathogenesis might play opposite roles in glioma development. These mechanisms are summarized herein. All of these mechanisms suggest that persons with schizophrenia would have lower risk of gliomas than persons without schizophrenia.

## The Similar Mechanisms Involved in Glioma Tumorigenesis and Schizophrenia

### Dysregulation of Schizophrenia-Related Genes in Glioma

#### DISC1

Disrupted-in-schizophrenia-1 (DISC1), a predisposing gene first found with its disruption by a balanced t(1;11) (q42;q14) in a large Scottish family, is identified to be closely related to major mental illnesses such as schizophrenia and bipolar disorder (St. Clair et al., 1990; Blackwood et al., 2001). Later, in a smaller American family with schizophrenia, the DISC1 gene was found to have a 4-bp frameshift mutation (Sachs et al., 2005). Studies further verified the potential association of the DISC1 gene with mental illnesses, especially schizophrenia, because many variants and polymorphisms of DISC1 were found in persons with schizophrenia (Thomson et al., 2013). Moreover, functional studies showed that DISC1 contributes to the etiopathology of mental illness, including regulating neural progenitor proliferation, neurite outgrowth, neuronal migration, synapse formation, neurogenesis and cAMP signaling (Lipina et al., 2012; Wen et al., 2014; Meng et al., 2016). These results demonstrated that DISC1 is strongly linked to the pathogenesis of schizophrenia.

Recently, our studies focused on the influence of DISC1 on glioma. A relatively higher expression of DISC1 was observed on glioma cells, and knocking down of DISC1 by shRNA significantly inhibited glioblastoma cell proliferation, migration, invasion and stem cell self-renewal. Further studies demonstrated that inhibition of DISC1 altered the mitochondrial dynamic by regulating Drp1 (Gao et al., 2016). Xie et al. (2015) showed that Drp1 activation is associated with poor prognosis of glioblastoma, suggesting that the mitochondrial dynamics were a regulatory switch for differentiation of glioma stem cells and could be treated as a new therapeutic target. Yin et al. (2016) proved that the Drp1 regulates glioma cell proliferation and invasion by the RHOA/ROCK1 pathway. Therefore, we first proposed a conclusion that DISC1 could play as an oncogene in GBM tumorigenesis.

Taken together, these data support the hypothesis that DISC1 might have the dual effect of regulating glioma tumorigenesis and neurodevelopment. It has been reported that DISC1 could interact with multiple proteins such as NDEL1, LIS1, GSK3β, 14-3-3, MAP1A, Girdin and PDE4 and play a role in either regulating neurodevelopment or promoting glioma progression (Gao et al., 2016).

#### P53

P53, one of the most important tumor suppressor genes, is usually lowly expressed in normal cells and regulates cellular stress responses (Hong et al., 2014). It has been reported that P53 is found to be mutated or deleted in more than 50% of all human cancers, suggesting that loss of P53 function contributes to cancer development (Peng et al., 2001; Olivier et al., 2002; Zheng et al., 2004; Hsu et al., 2010). Inhibition of P53 function helped to maintain the tumorigenic capacity of brain tumor-initiating cells (BTIC; Fukaya et al., 2016). Moreover, P53 mutant glioma patients exhibited therapeutic resistance and poor outcomes (Chen et al., 2017).

Recently, more and more studies have shown that P53 plays important roles in brain disorders (Agostini et al., 2018). P53 is highly expressed in the young mouse brain and lost in the adult mouse brain (Komarova et al., 1997; Ni et al., 2005). In 2000, Catts and Catts (2000) proposed that P53 might be a candidate susceptibility gene for schizophrenia by regulating apoptosis (Chiu et al., 2001). Moreover, the location of the P53 gene (chromosome 17p13.1) is close to 17p13.3, which is reported to have a significant linkage with schizophrenia incidence (Freedman et al., 2001). Ni et al. (2005) also reported a significant association between P53 and schizophrenia via case-control and family studies. Park et al. (2004) reported that the MspI polymorphisms of the P53 gene were specifically found in Korean schizophrenia and reduced the lung cancer predisposing. A similar result was found in the Turkish population by Ozbey et al. (2011). All these data suggest that P53 would be associated with schizophrenia and exhibit reduced vulnerability to cancer, but the detailed mechanism of the P53 gene in schizophrenia remains to be elucidated.

#### BDNF

Brain-derived neurotrophic factor (BDNF) plays an important role in neuronal development including neurogenesis, synaptic transmission and consolidation (Soule et al., 2006). Accumulating evidence indicates that the expression level of BDNF is altered in schizophrenia. Many studies showed that BDNF expression was reduced in persons with schizophrenia (Cannon et al., 2008; Pillai, 2008; Pandya et al., 2013), but some studies showed the opposite result (Reis et al., 2008). In addition, it has been reported that the epigenetic regulation of BDNF plays an important role in schizophrenia (Pandya et al., 2013). Kordi-Tamandani et al. (2012) showed that the downregulation of BDNF in persons with schizophrenia was associated with increased DNA methylation level of BDNF by methylation-specific PCR. In 2013, Tempei and colleagues reviewed the correlation between DNA methylation and BDNF promoter and found that the expression of BDNF in neural cells is tightly regulated by DNA methylation, thus suggesting a potential usefulness of the DNA methylation status of BDNF as a biomarker of psychiatric disorders including schizophrenia (Ikegame et al., 2013). Çöpoğlu et al. (2016) reported that there was a correlation between disease duration and DNA methylation, although there was no difference in methylation status of BDNF between persons with schizophrenia and controls. Furthermore, the polymorphisms of BDNF were reported to be associated with schizophrenia. The Val66Met polymorphism (rs6265) is a functional polymorphism affecting the secretion of BDNF. Sun et al. (2013) further found that the BDNF rs6265 may be a predisposing factor in schizophrenia from a Chinese Han population. A case-control meta-analysis showed a significant association between BDNF rs6265 and schizophrenia (Kheirollahi et al., 2016). All these data suggest that the altered BDNF function may be involved in schizophrenia pathophysiology (Angelucci et al., 2005).

In glioma patients, the expression of BDNF was significantly higher compared to control (Yan et al., 2009; Xiong et al., 2013b, 2015). Xiong et al. (2013a) found that the mature BDNF could promote glioma cell growth, inhibit cell apoptosis and increase cell motility and invasion. Furthermore, many studies reported that the Akt and Src are common downstream signaling kinase of BDNF and play an important role in glioma development (Sathornsumetee et al., 2007; Zhang et al., 2013; Saba et al., 2018). Inhibition of the Akt activation could inhibit the glioblastoma and glioblastoma stem-like cells (GSCs) growth, and induce apoptosis of malignant glioma cells (Gallia et al., 2009; Majewska and Szeliga, 2017; Shao et al., 2017). Src-family kinase (SFK) signaling is reported to affect a variety of tumor-related properties, particularly in the case of glioblastoma. Inhibition of Src could reduce growth and migration and change the motility of glioblastoma (Lewis-Tuffin et al., 2015). All these factors suggest that BDNF participates in the progression of glioma by regulating these oncogenic kinases.

#### CXCR4

C-X-C chemokine receptors type 4 (CXCR4) have been reported to overexpress in most cancers, including glioma (Gatti et al., 2013; Virgintino et al., 2013; Nazari et al., 2017). CXCL12 can bind to and activate CXCR4. Recent studies found that CXCR4 was significantly upregulated in GSCs and mediated the proliferation of GSCs. shRNA-mediated knockdown of CXCR4 *in vivo* also proved that CXCR4 could increase glioma perivascular invasion and reduce radiation-induced apoptosis (Yadav et al., 2016). PRX177561, a novel CXCR4 antagonist, could reduce GBM cell proliferation and accelerate GSC differentiation in preclinical models (Gravina et al., 2017). All these factors suggest a positive role of CXCR4 in glioma progression.

However, things seem different in schizophrenia. In the neuron development process, CXCR4 could regulate the migration and regional distribution of cortical interneurons (Li et al., 2008; Meechan et al., 2012). It has been reported that CXCR4 expression was downregulated and the promotor region of CXCR4 was hypermethylated in schizophrenia (Xu et al., 2012; Aberg et al., 2014). It has also been reported that 22q11 deletion syndrome (22q11DS) is a significant genetic predisposing factor for psychiatric conditions, including schizophrenia and bipolar disorder (Toritsuka et al., 2013). In a 22q11DS mouse model, the total CXCR4 protein reduced and the interneuron migration was disrupted (Meechan et al., 2012). All these factors suggest that the downregulation of CXCR4 in persons with schizophrenia would be a protective aspect for reducing the incidence of glioma.

The above examples illustrate that glioma tumorigenesis and schizophrenia may share similar mechanisms, and the molecular defects of DISC1, P53, BDNF and CXCR4 involved in schizophrenia pathogenesis might play opposite roles in glioma development (Table 2).

**Table 2:** List of genes and microRNAs (miRNAs) involved in glioma tumorigenesis and schizophrenia.

Genes/mRNAs	Function in schizophrenia	Function in glioma
DISC1	Common DISC1 variants are associated with schizophrenia	DISC1 acts as an oncogene in GBM tumorigenesis
p53	p53 might be a candidate susceptibility gene for schizophrenia	Loss of p53 function contributes to glioma development
BDNF	Altered BDNF function is involved in schizophrenia	Upregulation of BNDF participates in the progression of glioma
CXCR4	Downregulation of CXCR4 is associated with schizophrenia	CXCR4 plays a positive role in glioma progression
miR-9	Downregulation of miR-9 is associated with increased risk of schizophrenia	miR-9 is required for glioblastoma stem cell maintenance
miR-137	miR-137 is related to schizophrenia susceptibility	miR-137 acts as a tumor suppressor in glioma progression
miR-126	miR-126 is upregulated in the brain of persons with schizophrenia	miR-126 works as a tumor suppressor in glioma
miR-183	miR-183 is a protective biomarker for cancer in schizophrenic subjects	Upregulation of miR-183 promotes glioma progression

### Dysregulation of miRNA in Schizophrenia and Glioma

MicroRNAs (miRNAs or miRs) are small RNAs containing 20–23 nucleotides which regulate diverse biologic processes by inhibiting gene expression at the post-transcriptional level. Various studies have proved prominent roles of miRNAs in many physiological and pathological processes, and miRNAs could be a diagnostic and prognostic biomarker for various diseases such as cancer and CNS diseases including schizophrenia (Rao et al., 2013). Rizos et al. (2012) found that miR-183 was upregulated in the group of persons with schizophrenia without cancer but was almost undetectable in the group of persons with schizophrenia with a solid tumor. Therefore, they proposed that miR-183 could be a protective biomarker for cancer in schizophrenic subjects (Rizos et al., 2012). However, other studies indicated that highly expressed miR-183 promoted glioma cell proliferation and was significantly correlated with poor prognosis in glioma patients (Wang et al., 2016; Ye et al., 2016; Pavlakis et al., 2017). Therefore, whether miR-183 would be a protective gene for glioma in persons with schizophrenia needs to be further studied. MiR-9, another miRNA, was reported to be significantly downregulated in schizophrenia patient-derived neural progenitor cells, which affected neural migration and was associated with increased risk of schizophrenia (Topol et al., 2017). However, miR-9 was highly abundant in CD133(+) glioblastoma stem cells (Schraivogel et al., 2011). The Schizophrenia Psychiatric Genome-Wide Association Study (GWAS) Consortium (2011) reported that miR-137 was associated with schizophrenia susceptibility and regulated many schizophrenia susceptibility-related target genes (Yin et al., 2014). MiR-137 could also act as a tumor suppressor in glioma progression (Sun et al., 2015; Liang et al., 2016). It was reported that miR-126 was upregulated in the dorsolateral prefrontal cortex (DLPFC) of the brain in persons with schizophrenia (Beveridge et al., 2010). However, miR-126 worked as a tumor suppressor through inhibiting the target gene expression such as VEGF, CXCR4, DNMT1, KRAS in many cancers, especially glioma (Liu et al., 2015; Kong et al., 2016; Li et al., 2017). These studies indicated that many miRNAs play important roles in the development of glioma and schizophrenia (Table 1). The expression change of these miRNAs was associated with schizophrenia susceptibility but lower incidence of glioma.

### Antipsychotic Drugs Have Antitumor Effects

Many studies have proved that antipsychotic drugs may have antitumor and radiosensitizing effects (Carney et al., 2006; Fond et al., 2012). A systematic review in 2012 suggested that phenothiazines had antitumor effect by inhibiting various cancer proliferation (Fond et al., 2012). Olanzapine, a second-generation antipsychotic, has been shown to inhibit the human glioblastoma cell lines proliferation, migration, anchorage-independent growth and enhance TMZ anti-tumor activity (Karpel-Massler et al., 2015). Cheng et al. (2015) found that thioridazine, a widely used antipsychotic drug, had potent anti-GBM and anti-GSCs properties. Thioridazine could induce the autophagy and ER stress of the GBM cells and upregulate AMPK activity. More recently, Wang et al. (2017) proved that the atypical antipsychotic quetiapine (QUE) could suppress GSCs-initiated tumor growth, and the anti-tumor activity could be enhanced by combination with TMZ. Furthermore, QUE could induce the differentiation of GSCs towards OL-like cells by inhibiting the Wnt/β-catenin signaling pathway, and suppress GSC and TMZ-resistant gliomas initiation by affecting GSCs proliferation (Wang et al., 2017). ErbB and its ligand epidermal growth factor (EGF) are reported to participate in both glioma development and schizophrenia neuropathology (Berezowska and Schlegel, 2011; Sotoyama et al., 2011; Iwakura and Nawa, 2013; Brocard et al., 2015), so antipsychotic drug targeting the EGF/ErbBs signaling pathway can affect the tumorigenesis of glioma in persons with schizophrenia. Clozapine, an atypical antipsychotic drug that treated schizophrenia by blocking the EGF/ErbBs systems (Paulzen et al., 2014), has been reported to inhibit the U87MG human glioblastoma cells proliferation (Shin et al., 2006; Jeon et al., 2015). Many studies showed that chlorpromazine could inhibit the Akt/mTOR pathway and induce autophagic cell death in glioma cells (Shin et al., 2013), and inhibit the TMZ-resistant glioma cells growth and proliferation in orthotopic mouse brain tumor models (Oliva et al., 2017). Aripiprazole is also an atypical antipsychotic which is widely used to treat schizophrenia. Kim et al. (2018) recently proved that aripiprazole inhibits the glioma cell migration and induces apoptosis by directly inhibiting oncogenic Src kinase. It has been reported that the Src and Akt are common downstream signaling kinases of BDNF (Zhang et al., 2013), so these findings are consistent with the increase of BDNF in glioma patients. All these factors suggest that antipsychotic drugs have antitumor effects; therefore, when taking antipsychotic drugs, persons with schizophrenia also benefit from it inhibiting glioma development.

## Conclusions and Perspective

Several pieces of evidence provided here indicate that there might be a negative correlation between glioma and schizophrenia (Eskandari et al., 2015). Levav et al. (2007) found a reduced cancer risk among people with schizophrenia and proposed the presence of a gene with the dual effects of reducing cancer risk and disrupting neurodevelopment. We first discussed the genes which could play dual effects in glioma development and schizophrenia; the molecular defects of DISC1, P53, BDNF and CXCR4 involved in schizophrenia pathogenesis might play opposite roles in glioma development. Then, we indicated that many miRNAs such as miR-183, miR-9, miR-137 and miR-126 play important roles in the development of glioma and schizophrenia, and the expression change of these miRNAs was associated with schizophrenia susceptibility but lower incidence of glioma. Furthermore, many studies have found that antipsychotic drugs may have antitumor effects. All these studies showed that persons with schizophrenia have a decreased incidence of glioma, which can partially solve the “century puzzle” of whether persons with schizophrenia have a decreased incidence of cancer. Certainly, epidemiological investigation about glioma incidence and schizophrenia and further studies about the key genes/pathways involved in the pathogenesis of both glioma and schizophrenia, should be performed. These studies can provide more insightful understanding about glioma and schizophrenia pathophysiology and promote strategies for prevention and treatment applications in schizophrenia as well as cancer.

## Author Contributions

All authors listed have made substantial, direct and intellectual contribution to the work, and approved it for publication.

## Conflict of Interest Statement

The authors declare that the research was conducted in the absence of any commercial or financial relationships that could be construed as a potential conflict of interest.
